# Characteristics of health workers with COVID-19 at the beginning of the third wave in a national referral institute

**DOI:** 10.17843/rpmesp.2022.392.10749

**Published:** 2022-05-18

**Authors:** Zulema Tomas-Gonzales, Mónica Mallma-Silva, Javier Alarcón-Santos, Augusto Racchumí-Vela, María Medina-Pflucker

**Affiliations:** 1 Dirección General, Instituto Nacional de Salud del Niño-San Borja, Lima, Peru. Dirección General Instituto Nacional de Salud del Niño-San Borja Lima Peru; 2 Equipo de Recursos Humanos, Instituto Nacional de Salud del Niño-San Borja, Lima, Peru. Equipo de Recursos Humanos Instituto Nacional de Salud del Niño-San Borja Lima Peru; 3 Sub Unidad de Investigación e Innovación Tecnológica, Instituto Nacional de Salud del Niño-San Borja, Lima, Peru. Sub Unidad de Investigación e Innovación Tecnológica Instituto Nacional de Salud del Niño-San Borja Lima Peru

To the editor. By the end of 2021, the percentage of fully vaccinated people (two doses) worldwide was over 50% ^1^; however, the number of cases increased due to the high transmission capacity of the omicron variant, without an increase in hospitalizations and deaths, which showed that the disease produced by this variant is less severe ^2^ ^,^ ^3^. Nevertheless, the Pan American Health Organization (PAHO) emphasized that infections and reinfections by Omicron can be lethal in some cases, such as in immunocompromised and unvaccinated patients, and encouraged countries to reinforce the protection of health personnel ^3^.

In Peru, the third COVID-19 wave began in December 2021 and by January 17, 2022, an estimated 78.6% of the population was fully vaccinated ^4^. At that time, vaccination coverage among health personnel reached 100% at the Instituto Nacional de Salud del Niño-San Borja (INSNSB) (a national referral center providing highly specialized surgical care) ^5^. However, there was a large increase in cases, which caused a lack of personnel in the first line of care and limited care for other diseases. Therefore, the aim of this report is to describe the characteristics of health workers with COVID-19 at the beginning of the third wave at INSNSB.

A cross-sectional study was conducted on a population of 897 health workers who had a positive result for COVID-19. A total of 321 health workers were selected by simple random sampling. The parameters for estimating the sample size were based on an infection proportion of 70.3%, an error of 0.05 and a confidence level of 95%, using the formula of proportions for an infinite population. Screening was carried out with antigenic (immunochromatography) or molecular (polymerase chain reaction) testing, between December 22, 2021 and January 27, 2022. We analyzed the following variables: age, sex, professional service, type of test performed, condition, comorbidity, symptomatology and reason for test request (contact with confirmed case, contact with suspected case and prioritized).

Contact with a confirmed case was defined as having a contact with a positive test; contact with a suspected case was defined as having a contact with symptoms but without a confirmatory test, and a prioritized case was defined as a worker who was symptomatic but without a contact. The data were obtained from the epidemiological files collected by the INSNSB occupational health area. To analyze the data, we applied descriptive statistics using frequency tables and percentages, and measures of central position and dispersion.

Table 1 shows the main results; the highest proportion of infected persons were female (75.1%), the median age was 37 years (IQR:33 - 42), and the healthcare personnel was the group with the highest infection rate (78.5%). Of the cases, 95.3% presented symptoms, mostly sore throat (33.6%), nasal congestion (20.4%) and general malaise (16.1%). We found that 5.3% of those infected had some comorbidity, most frequently hypertension and asthma. Of the infected cases, 57.9% had some contact with a confirmed case, either in the family or at work; in addition, 45.2% had been discharged from the hospital and returned to work.

**Table 1:** Characteristics of COVID-19 cases from December 2021 to January 2022 at the Instituto Nacional de Salud del Niño-San Borja.

Characteristics	Total	
	n=321	%
Sex		
Female	241	75.1
Male	80	24.9
Age		
Median (IQR)	37 (33-42)	
Professional service		
Healthcare personnel	252	78.5
Administrative personnel	69	21.5
Type of test		
Antigen	315	98.7
Molecular	6	1.9
Condition		
Isolated	176	54.8
Discharged	145	45.2
Comorbidity		
Yes	17	5.3
No	304	94.7
Reason for test request		
Confirmed	186	57.9
Suspected	59	18.4
Prioritized	76	23.7
Symptomatic		
Yes	306	95.3
No	15	4.7
Symptoms		
Sore throat	255	33.6
Nasal congestion	155	20.4
General malaise	122	16.1
Cough	114	15.0
Fever/chills	84	11.1
Pain	21	2.8
Diarrhea	8	1.1

IQR: Interquartile range

This study describes the characteristics of INSNSB workers at the beginning of the third wave; no severe cases were reported. Similar situations were described in other hospitals in Peru, where the lack of personnel (due to medical leave) caused greater work pressure on front-line workers. Consequently, the Ministry of Health (MINSA) prepared a response plan based on updating technical standards. The National Health Council and regional governments were convened to coordinate a comprehensive response, and sectoral working groups as well as strategic intelligence groups were created; 750 health professionals were hired to work on the front line of the pandemic “COVID positions” and more than 5000 health workers were trained ^6^.

A limitation of this study is that many epidemiological files had incomplete data; however only 5% of the records had this issue, so they were excluded from the study.

In conclusion, the number of COVID-19 cases among health workers at INSNSB increased when the third wave started, with no severe cases. This allowed for a timely reinsertion into the workforce, which did not affect the care of pediatric patients. We recommend continuing with the supervision, monitoring, and evaluation of the COVID-19 response plan in the event of a possible fourth wave.
