# Supplementary material for: Characteristics of health workers with COVID-19 at the beginning of the third wave in a national referral institute
Source: Rev Peru Med Exp Salud Publica. 2022 May 18;39(2):245–6. doi: 10.17843/rpmesp.2022.392.10749 (PMC11397740; doi:10.17843/rpmesp.2022.392.10749)
Supplement: Translation

# Translation: Características de los trabajadores de salud con COVID-19 a inicios de la tercera ola en un instituto de referencia nacional

**Authors:** Zulema Tomas-Gonzales, Mónica Mallma-Silva, Javier Alarcón-Santos, Augusto Racchumí-Vela, María Medina-Pflucker

**Affiliations:** 1 Dirección General, Instituto Nacional de Salud del Niño-San Borja, Lima, Perú.; 2 Equipo de Recursos Humanos, Instituto Nacional de Salud del Niño-San Borja, Lima, Perú.; 3 Sub Unidad de Investigación e Innovación Tecnológica, Instituto Nacional de Salud del Niño-San Borja, Lima, Perú.

Sr. Editor. En el mundo, a finales del 2021, el porcentaje de personas con vacunación completa (dos dosis) fue superior al 50% ^1^; sin embargo, se observó un aumento de casos debido a la alta capacidad de contagio de la variante ómicron, sin un incremento de hospitalizaciones y muertes; esto demostró una menor gravedad de la enfermedad causada por esta variante ^2^ ^,^ ^3^. No obstante, la Organización Panamericana de la Salud (OPS) resaltó que las reinfecciones e infecciones por ómicron pueden ser letales en algunos casos, como en inmunodeprimidos y en no vacunados, y alentó a los países a reforzar la protección del personal de salud ^3^.

La tercera ola en el Perú se inició en diciembre de 2021 y hasta el 17 de enero de 2022 se estimó que el 78,6% de la población tenía la vacunación completa ^4^. En ese momento, en el Instituto Nacional de Salud del Niño-San Borja (INSNSB) (centro de referencia nacional que brinda atención quirúrgica altamente especializada), la cobertura de vacunación en el personal de salud alcanzó el 100% ^5^. Sin embargo, se observó un gran aumento de casos, lo que ocasionó bajas en la primera línea de atención y limitó la atención de otras enfermedades. Por ello, el objetivo de este reporte es describir las características de los trabajadores de salud con COVID-19 a inicios de la tercera ola, en el INSNSB.

Se realizó un estudio transversal sobre una población de 897 trabajadores de salud con resultado positivo a la COVID-19. Se seleccionaron 321 trabajadores mediante muestreo aleatorio simple. Los parámetros para la estimación del tamaño de muestra se basaron en una proporción de infección de 70,3%, error de 0,05 y un nivel de confianza del 95%, usando la fórmula de proporciones para una población infinita. El tamizaje fue realizado con prueba antigénica (inmunocromatografía) o molecular (reacción en cadena de la polimerasa), entre el 22 de diciembre de 2021 al 27 de enero del 2022. Las variables analizadas fueron: edad, sexo, servicio profesional, tipo de prueba realizada, condición, comorbilidad, sintomatología y motivo de solicitud de prueba (contacto con caso confirmado, contacto con caso sospechoso y priorizados). 

El contacto con caso confirmado se definió cuando el contacto tenía prueba positiva; el contacto con caso sospechoso se definió cuando el contacto tenía síntomas, pero sin prueba confirmatoria, en tanto que el priorizado fue definido como aquel trabajador que estaba sintomático, pero sin contacto detectado. Los datos se obtuvieron de las fichas epidemiológicas colectadas por el área de salud ocupacional del INSNSB. Para el análisis de datos se utilizó estadística descriptiva mediante tablas de frecuencias y porcentajes, y medidas de posición central y dispersión. 

La Tabla 1 muestra los principales resultados del análisis; la mayor proporción de infectados fueron del sexo femenino (75,1%), la mediana de la edad fue de 37 años (RIC:33 - 42), y el personal asistencial fue el grupo con mayor contagio (78,5%). El 95,3% de los casos presentaron síntomas, mayormente dolor de garganta (33,6%), congestión nasal (20,4%) y malestar general (16,1%). Se encontró que un 5,3% de los infectados tenía alguna comorbilidad, con mayor frecuencia hipertensión y asma. El 57,9% de los casos de infección tuvieron algún contacto con un caso confirmado, ya sea familiar o laboral; además, el 45,2% se encontraba de alta médica, y retornaron a sus actividades laborales.

**Tabla 1:** Características de los casos de COVID-19 de diciembre de 2021 a enero de 2022 en el Instituto Nacional de Salud del Niño-San Borja.

Características	Total	
	n=321	%
Sexo		
Femenino	241	75,1
Masculino	80	24,9
Edad		
Mediana (RIC)*	37	(33 - 42)
Servicio profesional		
Personal asistencial	252	78,5
Personal administrativo	69	21,5
Tipo de prueba		
Antígeno	315	98,1
Molecular	6	1,9
Condición		
Aislamiento	176	54,8
Alta	145	45,2
Comorbilidad		
Sí	17	5,3
No	304	94,7
Motivo de solicitud de prueba		
Confirmado	186	57,9
Sospechoso	59	18,4
Priorizado	76	23,7
Sintomático		
Sí	306	95,3
No	15	4,7
Síntomas		
Dolor de garganta	255	33,6
Congestión nasal	155	20,4
Malestar general	122	16,1
Tos	114	15,0
Fiebre/escalofríos	84	11,1
Dolor	21	2,8
Diarrea	8	1,1

* RIC: rango intercuartílico

El estudio describe las características de los trabajadores del INSNSB a inicios de la tercera ola; sin presentarse casos graves. Situaciones similares se describieron en otros centros hospitalarios del Perú, en donde la falta de personal (debido a la licencia por descanso médico), ocasionó una mayor presión laboral en los trabajadores de primera línea. En consecuencia, el Ministerio de Salud (MINSA) formuló un plan de respuesta basado en actualización de las normas técnicas, se convocó al Consejo Nacional de Salud y a los gobiernos regionales para coordinar una respuesta integral, se crearon grupos de trabajo sectorial y de inteligencia estratégica, se contrató 750 profesionales sanitarios para trabajar en la primera línea de la pandemia “plazas COVID” y se capacitó a más de 5000 trabajadores de salud ^6^.

Se debe reconocer como una limitación del estudio que muchas fichas epidemiológicas tenían registros incompletos; sin embargo, esto solo representó el 5% de los casos, por lo que fueron excluidos del estudio.

En conclusión, a inicios de la tercera ola, en el INSNSB se incrementó el número de casos de COVID-19 en los trabajadores de salud, sin presentarse casos graves. Esto permitió una reinserción laboral oportuna, que no afectó la atención de los pacientes pediátricos. Se recomienda continuar con las acciones de supervisión, monitoreo y evaluación del plan de respuesta frente a la COVID-19, ante una posible cuarta ola.
